# Presence of multi-drug resistant pathogenic *Escherichia coli* in the San Pedro River located in the State of Aguascalientes, Mexico

**DOI:** 10.3389/fmicb.2013.00147

**Published:** 2013-06-17

**Authors:** Flor Y. Ramírez Castillo, Francisco J. Avelar González, Philippe Garneau, Francisco Márquez Díaz, Alma L. Guerrero Barrera, Josée Harel

**Affiliations:** ^1^Laboratorio de Biología Celular y Tisular, Departamento de Morfología, Centro de Ciencias Básicas, Universidad Autónoma de AguascalientesAguascalientes, México; ^2^Laboratorio de Ciencias Ambientales, Departamento de Fisiología y Farmacología, Centro de Ciencias Básicas, Universidad Autónoma de AguascalientesAguascalientes, México; ^3^Faculté de Médecine Vétérinaire, Centre de Recherche en Infectiologie Porcine, Université de Montréal, St-HyacintheQC, Canada; ^4^Departamento de Infectología, Centenario Hospital Miguel HidalgoAguascalientes, México

**Keywords:** water quality, antibiotic resistance, fluoroquinolone, virulence factors, pathogenic *Escherichia coli*

## Abstract

Contamination of surface waters in developing countries is a great concern. Treated and untreated wastewaters have been discharged into rivers and streams, leading to possible waterborne infection outbreaks and may represent a significant dissemination mechanism of antibiotic resistance genes. In this study, the water quality of San Pedro River, the main river and pluvial collector of the Aguascalientes State, Mexico was assessed. Thirty sample locations were tested throughout the River. The main physicochemical parameters of water were evaluated. Results showed high levels of fecal pollution as well as inorganic and organic matter abundant enough to support the heterotrophic growth of microorganisms. These results indicate poor water quality in samples from different locations. One hundred and fifty *Escherichia coli* were collected and screened by PCR for several virulence genes. Isolates were classified as either pathogenic (*n* = 91) or commensal (*n* = 59). The disc diffusion method was used to determine antimicrobial susceptibility to 13 antibiotics. Fifty-two percent of the isolates were resistant to at least one antimicrobial agent and 30.6% were multi-resistant. Eighteen *E. coli* strains were quinolone resistant of which 16 were multi-resistant. Plasmid-mediated quinolone resistance (PMQR) genes were detected in 12 isolates. Mutations at the Ser-83→Leu and/or Asp-87→Asn in the *gyrA* gene were detected as well as mutations at the Ser-80→Ile in *parC*. An *E. coli* microarray (Maxivirulence V 3.1) was used to characterize the virulence and antimicrobial resistance genes profiles of the fluoroquinolone-resistant isolates. Antimicrobial resistance genes such as *bla*_TEM_, *sulI*, *sulII*, *dhfrIX*, *aph3* (*str*A), and *tet* (B) as well as integrons were found in fluoroquinolone (FQ) resistance *E. coli* strains. The presence of potential pathogenic *E. coli* and antibiotic resistance in San Pedro River such as FQ resistant *E. coli* could pose a potential threat to human and animal health.

## Introduction

*Escherichia coli* is a commensal member of the intestinal flora of humans and warm-blooded animals. Several genotypes have acquired specific virulence factors and are capable of causing disease as gastrointestinal diseases, urinary tract infections (UTIs) and sepsis/meningitis. Intestinal pathogenic strains causing diarrhea include enteropathogenic *E. coli* (EPEC), enterohemorragic *E. coli* (EHEC), enterotoxigenic *E. coli* (ETEC), enteroaggregative *E. coli* (EAEC), enteroinvasive *E. coli* (EIEC) and diffusely adherent *E. coli* (DAEC, Nataro and Kaper, 1998). The extraintestinal pathogenic *E. coli* (ExPEC) group is composed of uropathogenic *E. coli* (UPEC), which is the main causes of UTIs, meningitis-associated *E. coli* (MNEC), sepsis-associated *E. coli* (SEPEC) and the avian pathogenic *E. coli* (APEC), which is associated with respiratory infections, pericarditis, and septicaemia in poultry (Kaper et al., 2004).

The presence of *E. coli* in water is widely used as a microbiological indicator of fecal pollution and water quality (WHO, 2006). The presence of pathogenic *E. coli* in environmental water creates a potential risk for infections in humans and animals especially since water is used for irrigation, a source of drinking water, and for recreational purposes (Hamelin et al., 2006, 2007; Kümmerer, 2009a; Koczura et al., 2012). Furthermore, the presence of antimicrobial resistant bacteria in the environment is another great concern for public health.

The contamination of aquatic and soil environments by untreated sewage or manure coming from human or animals treated with antibiotics (Baquero et al., 2008; Kümmerer, 2009b; Lupo et al., 2012; Suzuki and Hoa, 2012), as well as the presence of heavy metal pollution (McArthur and Tuckfield, 2000) and quaternary ammonium compounds (QACs; Hegstad et al., 2010) might result in the presence of antimicrobial resistant bacteria. Furthermore, most antibiotics are not fully eliminated during the sewage treatment process. Thus, an aquatic environment such as rivers or streams could act as an antibiotic resistant genes reservoir and facilitate the dissemination of these genes (Kümmerer, 2004; Lupo et al., 2012). The emergence of antimicrobial resistance mechanisms, especially those associated with mobile genetic elements, may enhances the possibility that virulence factors genes and antibiotic resistance genes are spread simultaneously, inducing the emergence of new pathogens (Chen et al., 2011; Da Silva and Mendoça, 2012; Koczura et al., 2012).

Fluoroquinolone (FQ) is a family of widely used synthetic antimicrobial agents with a broad antibacterial spectrum that is used as a front line drug for urinary tract and intestinal infections. However, increase in the prevalence of FQ resistant bacteria has been a great concern worldwide in the last years. Several mechanisms have been described for FQ resistance. In *E. coli*, the resistance is primarily associated with the accumulation of mutations in the quinolone-resistance determining regions (QRDRs) of *gyrA* and *parC*, which encode topoisomerase II (DNA gyrase) and topoisomerase IV respectively (Hooper, 2001; Hopkins et al., 2005). These mutations can lead to conformational changes in the enzymes and thus preventing quinolones from binding to the DNA-substrate complex (Tran et al., 2005a,b). In addition, several other mechanisms can contribute to FQ resistance, including plasmid-mediated quinolone resistance (PMQR) determinants (Martinez-Martinez et al., 1998), such as the Qnr protein (QnrA, QnrB, QnrC, QnrD, and QnrS), the variant of the aminoglycoside-modifying enzyme, AAC(6′)-Ib-cr (Robicsek et al., 2006a), and the efflux pumps QepA (Yamane et al., 2007), and OqxAB (Hansen et al., 2004; Jacoby, 2005).

Pollution of surface waters may represent a pathway for the global dissemination of antibiotic resistance (Chen et al., 2011). Water contamination is a major environmental problem in Mexico. In the last decades, treated, and untreated wastewater have been released into natural streams leading to possible waterborne disease outbreaks resulting from the use of unsafe water for consumption, irrigation or recreational activities (Mazari-Hiriart et al., 2008; Chávez et al., 2011). Several studies on antibiotic resistant bacteria release and their occurrence in sewage and natural environments have been conducted (Sayah et al., 2005; Amábile-Cuevas et al., 2010; Chen et al., 2011; West et al., 2011; Czekalski et al., 2012; Sun et al., 2012; Tacão et al., 2012). However, the significance of rivers as environments providing irrigation water and recreational activities as well as the possibility of the potential spread of pathogenic and antibiotic resistant bacteria between the environment and humans and animals, marks them as highly relevant study subjects (Czekalski et al., 2012). In addition, anthropogenic activities might impact on water environment promoting the dissemination of antibiotic resistance (Pruden et al., 2006; Martinez, 2009; Tacão et al., 2012). Thus, it is of major importance to study how human activities can impact antimicrobial resistant bacteria and antimicrobial resistance genes in the environment in order to understand their spread and health implications (Tacão et al., 2012).

In this study, the water quality of San Pedro River of Aguascalientes State in Mexico was evaluated. Currently, the river is being contaminated by wastewater from urban municipal sewers, industrial activities, and livestock farms. The objectives of this study were to evaluate the quality of the water between sample locations in the river polluted by different human activities, to determine the presence of pathogenic *E. coli* and assess the antimicrobial resistance profiles for the *E. coli* isolates, focusing in FQs agents.

## Materials and methods

### Water sampling and *E. coli* isolation

**Figure 1:**
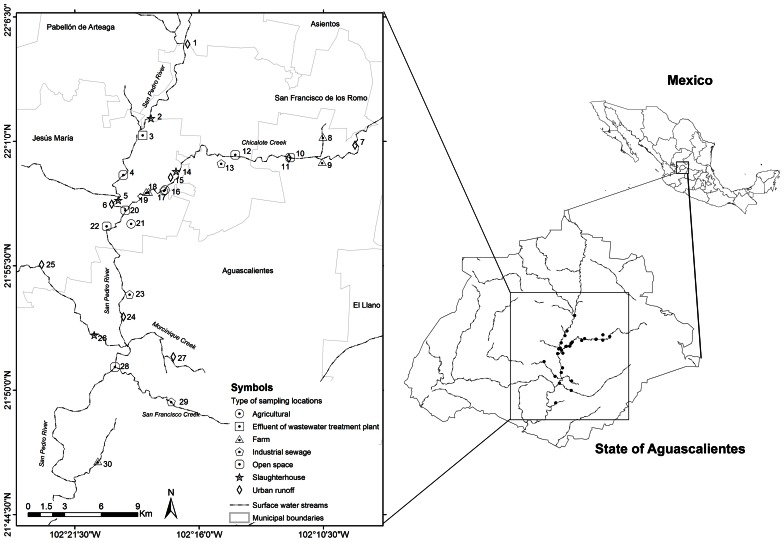
**Diagram of sample locations along the San Pedro River**. Chicalote, Morcinique, and San Francisco Creek are the main tributaries of the San Pedro River.

Thirty sample locations were selected throughout San Pedro River and its main tributaries (Figure 1). All locations were sampled once and selected due to the presence of important discharges of treated or untreated wastewater into the river (Table 1). San Pedro River is the main watershed and pluvial collector of Aguascalientes State. The river belongs to the hydrological region of Lerma-Santiago-Pacifico which is one of the most important drainage basin of Mexico (77,000 km^2^), draining 80% of the Aguascalientes State. Currently, the San Pedro River is being contaminated by the influx of wastewater from population centers, industrial activities, agricultural activities, and livestock which results in different levels of surface water quality. It is estimated that ~60% of the sewage water is discharged into the river without prior treatment (CONABIO, 2008). Nevertheless, the river is still used as important source for agricultural irrigation as well as recreational purposes in the cleanest zones (Gúzman-Colis et al., 2011).

**Table 1:** **Location and main sources of pollution of the 30 sampling locations studied**.

**Sample no**.	**Sample locations**	**^a^Main source of pollution**	**^b^Type of sampling location**
1	San Pedro River. Las Animas.	Urban runoff	UR
2	San Pedro River. FREASA	Slaughterhouse	S
3	San Pedro River. FREASA-PT.	Wastewater treatment plant effluent	WWTP-E
4	San Pedro River. San Antonio de los Horcones.	Open space	OS
5	San Pedro River. Rastro municipal de Jesus Maria.	Slaughterhouse	S
6	San Pedro River. Los Ramiez-Los Vasquez.	Human sewage + wastewater discharge	UR
7	Chicalote Creek. Jaltomate.	Urban runoff	UR
8	Chicalote Creek. El Becerro II.	Agricultural + farm	F
9	Chicalote Creek. El Becerro I.	Agricultural + farm	F
10	Chicalote Creek. Cañada Honda I.	Urban runoff	UR
11	Chicalote Creek. Cañada Honda II.	Open space	OS
12	Chicalote Creek. Loretito.	Open Space	OS
13	San Pedro-Chicalote River. Confluencia PIVA-Chicalote-Gomez Portugal.	Urban runoff + agricultural + wastewater + industrial sewage	IS
14	Chicalote Creek. Gomez Portugal - Area oeste.	Urban runoff + agricultural + wastewater	UR
15	Chicalote Creek. Gomez Portugal.	Agricultural + slaughterhouse	S
16	Chicalote Creek. Reserva Brandy.	Urban runoff + agricultural	AR
17	San Pedro-Chicalote River. Confluencia PIVA-Chicalote.	Urban runoff + agricultural + wastewater + industrial sewage + slaughterhouses	IS
18	Chicalote Creek. La Florida I.	Agricultural + farm	F
19	Chicalote Creek. La Florida II.	Agricultural + farm	F
20	San Pedro River-Chicalote Creek.	Open space	OS
21	San Pedro River. Paso Blanco.	Agricultural	AR
22	San Pedro River. Tepetate-San Miguelito.	Open space	OS
23	San Pedro River. Canal interceptor.	Urban runoff + wastewater discharge + industrial sewage	IS
24	San Pedro River. Puente Curtidores.	Urban runoff + wastewater discharge	UR
25	Morcinique Creek. Los Arquitos.	Urban runoff + agricultural + wastewater	UR
26	Morcinique. Los Negritos primera seccion.	Slaughterhouse	S
27	San Pedro River-Morcinique Creek. Aguascalientes Lopez Portillo.	Urban runoff	UR
28	San Pedro River-San Francisco Creek.	Open space	OS
29	San Pedro River-San Francisco Creek. Puente Bonaterra.	Industrial sewage	IS
30	San Pedro River. Fatima.	Agricultural + farm + human sewage	F

a Main source of pollution of location was determined based on the presence of discharge nearby and the type of land activity.

b AR, agriculturally impaired; F, farm; IS, industrial sewage; OS, open spaces; S, slaughterhouse; UR, urban runoff; WWTP-E, wastewater treatment plant effluent.

Sampling was performed from June to November according to procedure described by the American Public Health Association (APHA, 1998) and the World Health Organization (WHO, 2006; Table 1). Water samples were collected in 180 mL sterile glass bottles in triplicates. The samples were stored at 4°C until analysis, which was done within 24 h of the sample collection. To isolate *E. coli*, serial dilutions of the sample were prepared in 0.85% NaCl, and were used to inoculate MacConkey agar and Eosin Methylene Blue medium agar. Plates were incubated overnight at 37°C. For further identification, possible *E. coli* were confirmed by testing for glucuronidase activity (growth and fluorescence in EC-MUG), citrate utilization, indole production, methyl red test and Voghes–Proskauer test. Isolates meeting the *E. coli* test profile were confirmed by detecting the *uidA* gene using *uidA* primers listed in Table 2. The *E. coli* isolates were stored at −80°C in tryptic soy broth and 20% (vol/vol) glycerol.

**Table 2:** **Oligonucleotides used in this study**.

**Oligonucleotide name**	**Target gene**	**Oligonucleotide 5′ → 3′**	**Amplification product (bp)**	**Reference**
***E. coli*** MARKER				
uidA-forward	*uidA*	ATGTGCTGTGCCTGAACC	450	This study.
uidA-reverse		ATTGTTTGCCTCCCTGCTG		
**VIRULENCE GENES FOR INTESTINAL PATHOGENIC *E. coli***				
VTcom-forward	*stx1/stx2*	GAGCGAAATAATTTATATGTG	518	Toma et al., 2003
VTcom-reverse		TGATGATGGCAATTCAGTAT		
East-forward	*eastI*	ATGCCATCAACACAGTATAT	110	Vila et al., 2000
East-reverse		GCGAGTGACGGCTTTGTAGT		
AafAf	*aafA*	AAATTAATTCCGGCATGG	518	Huang et al., 2007
AafAr		ATGTATTTTTAGAGGTTGAC		
aggRks1	*aggR*	GTATACACAAAAGAAGGAAGC	254	Aranda et al., 2007
aggRksa2		ACAGAATCGTCAGCATCAGC		
AL65	*est*	TTAATAGCACCCGGTACAAGCAGG	147	Toma et al., 2003
Al125		CCTGACTCTTCAAAAGAGAAAATTAC		
LTL	*elt*	TCTCTATGTGCATACGGAGC	322	Toma et al., 2003
LTR		CCATACTGATTGCCGCAAT		
IpaIII	*ipaH*	GTTCCTTGACCGCCTTTCCGATACCGTC	619	Toma et al., 2003
IpaIV		GCCGGTCAGCCACCCTCTGAGAGTAC		
BFP1	*bfpA*	AATGGTGCTTGCGCTTGCTGC	326	Aranda et al., 2007
BFP2		GCCGCTTTATCCAACCTGGTA		
eae1	*eae*	CTGAACGGCGATTACGCGAA	880	Aranda et al., 2007
eae3		CGAGACGATACGATCCAG		
**VIRULENCE GENES FOR EXTRA-INTESTINAL PATHOGENIC *E. coli***				
papC-forward	*papC*	GACGGCTGTACTGCAGGGTGTGGCG	350	Blanco et al., 1997
papC-reverse		ATATCCTTTCTGCAGGGATGCAATA		
SfaSf	*sfaS*	GTGGATACGACGATTACTGTG	240	Johnson and Stell, 2000
SfaSr		CCGCCAGCATTCCCTGTATTC		
Afaf	*afa/dra*	GGCAGAGGGCCGGCAACAGGC	592	Johnson and Stell, 2000
Afar		CCCGTAACGCGCCAGCATCTC		
FyuAf	*fyuA*	TGATTAACCCCGCGACGGGAA	880	Johnson and Stell, 2000
FyuAr		CGCAGTAGGCACGATGTTGTA		
KpsMIIf	*kpsMT II*	GCGCATTTGCTGATACTGTTG	272	Johnson and Stell, 2000
KpsMIIr		CATCCAGACGATAAGCATGAGCA		
**QUINOLONE RESISTANCE GENES**				
gyrA11753	*gyrA*	GTATAACGCATTGCCGC	251	Wang et al., 2001
gyrA12004		TGCCAGATGTCCGAGAT		
EC-PAR-A	*parC*	CTGAATGCCAGCGCCAAATT	189	Deguchi et al., 1997
EC-PAR-B		GCGAACGATTTCGGATCGTC		
qnrA-forward	*qnrA*	TCAGCAAGAGGATTTCTCA	605	Maynard et al., 2004
qnrA-reverse		GGCAGCACTATTACTCCCA		
qnrB-forward	*qnrB*	GATCGTGAAAGCCAGAAAGG	469	Robicsek et al., 2006b
qnrB-reverse		ACGATGCCTGGTAGTTGTCC		
qnrS-forward	*qnrS*	ACGACATTCGTCAACTGCAA	417	Robicsek et al., 2006b
qnrS-reverse		TAAATTGGCACCCTGTAGGC		
aac-forward	*acc-(6′)-lb*	TTGCGATGCTCTATGAGTGGCTA	482	Park et al., 2006
aac-reverse		CTCGAATGCCTGGCGTGTTT		

### Physicochemical parameters

All techniques were performed according to Standard Methods (APHA, 1998). Water temperature (Method 2550B), pH (Method 4500 – H + B), conductivity (2510B) and dissolved oxygen (4500 OG) were determined electrometrically *in situ*. The level of organic matter pollution was determined using the biological oxygen demand (BOD, 5210B) and chemical oxygen demand (COD, 5220D). Total nitrogen (4500–NorgB), total phosphorus (4500–PE) and total suspended solids (TSS) (2540B–F) were also determined.

### Microbiological analysis

The amount of total bacteria was estimated by testing for mesophilic microorganisms using the pour plates technique (9215B). Fecal contamination was determined by measuring total coliform (9221C) and fecal coliform (9221E) using the standard fermentation technique for the most probable number (MPN; APHA, 1998).

### Antimicrobial susceptibility testing

Antimicrobial susceptibility test of 150 *E. coli* isolated from stream water was performed using a disc diffusion assay according to CLSI standards, 2010. Isolates that were resistant to three or more antimicrobial agents were defined to have a multiple drug resistant (MDR) phenotype. *E. coli* ATCC 25922 (American Type Culture Collection, Manassas, VA, US), was included in each assay as a negative control. Antimicrobial agents were tested using a Bio-Rad (Hercules, CA, US) Sensi–Disc antimicrobial susceptibility test multidisc for Gram–negative bacteria with the following antimicrobial agents: amikacin-30, ampicillin-10, cephalothine-30, cephotaxim-30, ceftriaxone-30, chloramphenicol-30, gentamicin-10, netilmicin-30, nitrofurantoin-300, pefloxacin-5, carbenicillin-30, and trimethoprim-sulfamethoxazole-1.25/23.75 μg. The quinolone levofloxacin-5 μg was tested separately using a Bio-Rad (Mexico, DF, Mexico) Sensi-Disc.

### DNA extraction

*E. coli* isolates were grown overnight in 5 mL of Luria–Bertani broth at 37°C without shaking. An aliquot (1 mL) of the overnight culture was transferred to 1.5 mL tubes and centrifuged at 15,500 × *g* for 2 min. The supernatant was removed, and the cell pellet was resuspended by vortexing in 200 μL of sterile water. The suspension was boiled for 15 min, centrifuged (15,500 × *g*, 2 min), and 150 μL of the supernatant was collected (Hamelin et al., 2006). The DNA quality and purity extracted was analyzed by spectrophotometer at 260 and 280 nm wavelengths. The sample with ratio (λ_260_/λ_280_) ≥ 1.5 was considered adequate to continue with identification by polymerase chain reaction (PCR).

### Pathotype and virulence genes determination by PCR

Detection of intestinal pathogenic *E. coli* (InPEC) and ExPEC virulence genes were performed by PCR with primers described in Table 2. InPEC isolates were defined according to criteria established by Aranda et al., 2007 and ExPEC isolates were defined by criteria described by Johnson and Stell, 2000. Positive controls are listed in Table 3.

**Table 3:** **PCR control strains used in this study**.

**Strain**	**Positive gene (s)**
ETEC H10407	*elt* and *est*
EHEC EDL933	*sxt1* and *sxt2*
EPEC 2349/69	*eae* and *bfpA*
EAEC O42	*aggR* and* aaf genes*
*Shigella flexneri*	*ipaH*
J53pMG252	*qnrA*
J53pMG298	*qnrB*
J53pMG306	*qnrS*
*Salmonella* SA20042859	*aac(6′)-Ib*

### Chromosomal-encoded and acquired quinolone resistance genes

Eighteen quinolone resistant and intermediate resistant isolates were selected to investigate mutations in the QRDR of *gyrA* and *parC* genes, as well as the presence of the acquired genes *qnrA*, *qnrB*, *qnrS*, and *aac (6′)-Ib cr*. The oligonucleotides and PCR conditions used in this study are listed in Table 2. The quinolone resistance determining regions of *gyrA* and *parC* genes were amplified and sequenced as described by Namboodiri et al. (2011). Amplicons were sequenced on both strands and predicted peptide sequences were compared to the corresponding gene from the MG1655 genome using BLAST program in Geneious R6 software (v. 6.0., Biomatters Ltd., New Zealand). The strains J53pMG252, J53pMG298, and J53pMG306 were used as positive controls for *qnrA*, *qnrB*, and *qnrS*, respectively (Jacoby et al., 2003) and water was used as negative control. The *aac (6′)-Ib cr* genes was detected as described by Park et al. (2006) with some modifications. Primers were used as follows: aac-forward (5′-TTGCGATGCTCTATGAGTGGCTA-3′) and aac-reverse (5′-CTCGAATGCCTGCGCTGTTT-3′) to yield an amplicon of 482 bp. The PCR condition were 94°C for 4 min, 35 cycles of 94°C of 30 s, 58°C for 30 s, and 68°C for 45 s, and a final step of 68°C for 10 min. Positive controls are listed in Table 3. The *aac (6′)-Ib cr* variant was identified by sequencing the PCR products (Park et al., 2006).

### DNA microarray analysis

Microarray hybridizations were performed using *E. coli* Maxivirulence version 3.1 microarray as previously described (Jakobsen et al., 2011). It allows the detection of 348 virulence genes and 98 antibiotic resistance genes and variants. DNA extraction and hybridizations were performed as described previously (Bruant et al., 2006). Each isolate was assigned to a specific *E. coli* pathotype according to its virulence gene profile and based on classification described previously (Bonnet et al., 2009; Jakobsen et al., 2011). *E. coli* isolates were also assigned to a phylogenetic group based on the presence of *chuA*, *TspE4.C2*, and *yjaA* as described previously (Clermont et al., 2000).

### Statistical analysis

Comparisons of associations between resistant phenotypes in *E. coli* isolated from stream were performed separately by using Pearson's chi-square exact test and Fisher's exact test (STATISTICA V. 10, StatSoft, US). Spearman's rank correlation (West et al., 2011) was used to examine the relationships among temperature, pH, conductivity, dissolved oxygen, COD, BOD, total phosphorus, nitrogen, total solid suspended, mesophilic bacteria and total and fecal coliform density across all sample locations.

## Results

### Physicochemical analyses of San Pedro River

**Figure 2:**
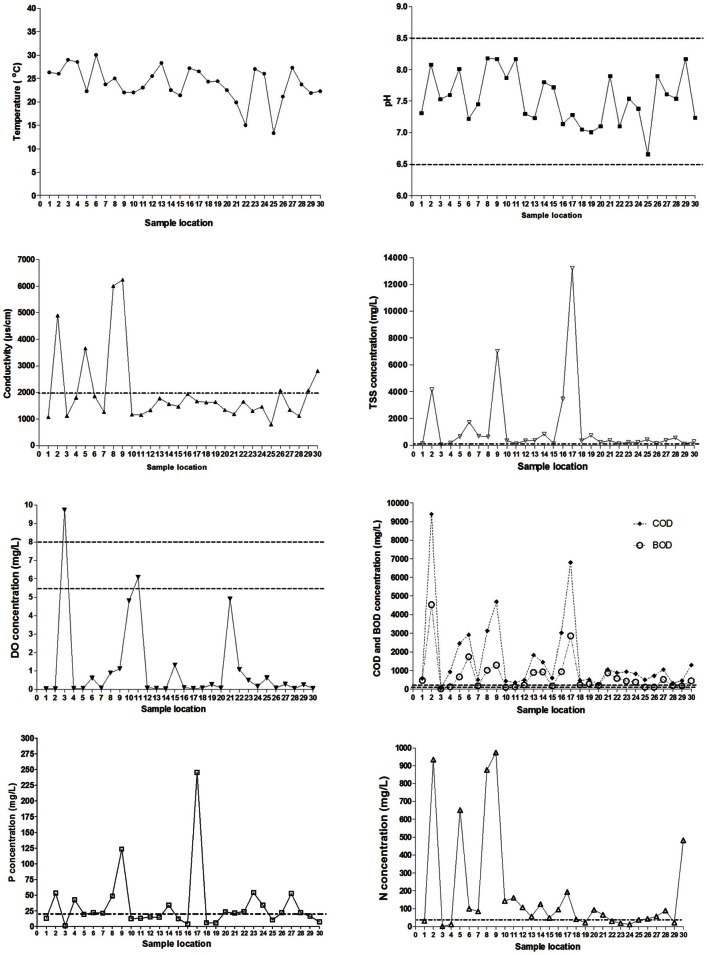
**Spatial variation of physico-chemical parameters from sampling locations on the San Pedro River**. Permissible maximum limits are shown in dotted lines. pH values between 6.5 and 8.5 units are considered as good quality in potable water (NOM-127-SSA1-1994). Conductivity values above 2000 μS/cm are exceeding the permissible limits. Nitrogen values above 40 mg/L are exceeding the permissible limits. Phosphorus levels rating higher than 20 mg/L is considered as poor water quality. Total solid suspended has a permissible value of 120 mg/L. Dissolved oxygen concentration limits are between 5.5 and 8.0 mg/L (Gúzman-Colis et al., 2011). BOD and COD concentrations limits are 150 and 100 mg/L respectively according to Mexican norms NOM-003-ECOL-1997 and NOM-001-ECOL-1996.

The physicochemical properties of each sample locations of the San Pedro River (Figure 1) were characterized using parameters such as temperature, DO, pH, BOD, and COD. These parameters were included because they have a major influence on bacterial growth (Salem et al., 2011). Results of physicochemical parameters are shown in the Figure 2. The mean value for water temperature was 24.2°C, which is usually considered a favorable temperature for the growth of microorganisms as well as for a wide range of human and animal pathogens. The pH values of water samples varied between 6.7 and 8.2 units, with a mean value of 7.6 units. The pH values were within the permissible limits (6.0–9.0) established by the WHO for wastewater discharge into the sea or the environment. Furthermore, this pH range value is optimal for bacterial growth.

The dissolved oxygen concentrations (DO) for almost all the sample locations were lower than 1 mg/L suggesting high organic matter levels. These DO concentrations are near anoxygenic conditions, which allow the growth of a broad spectrum of both aerobic and anaerobic microorganisms. For conductivity, 96.6% of the samples were above the WHO guideline value of 1000 μS/cm for wastewater discharging into a stream. These conductivity levels imply high concentrations of dissolved inorganic matter suggesting that high concentrations of inorganic nutrients are available for microbial proliferation. Significant differences in conductivity were observed between location sites close to farms and slaughterhouses (*p* < 0.001) compared to other types of sample locations.

The indicators of organic matter, COD and BOD, were generally high along sample locations (Figure 2). This confirms the discharge of raw wastewater of urban runoff including municipal origin into the river. The mean values were 722 and 1723 mg/L for BOD and COD, respectively, representing a high organic load in the river. Sample locations with highest levels of BOD and COD were related to farms, urban runoff, industrial sewage and slaughterhouses. All water samples were above the maximum permissible limit of organic matter (BOD < 200 mg/L) set by Mexican Norms (NOM-001-ECOL-1996) for streams discharged into rivers used for agricultural irrigation. These results showed abundant carbon and energy sources to support the heterotrophic growth of microorganisms.

Total solid suspended (TSS) were correlated with concentration of COD, BOD, and mesophilic bacteria [TSS–COD, *r*_*s*_ = 0.79, *p* = 0.05; TSS–BOD, *r*_*s*_ = 0.79, *p* = 0.05; TSS–mesophilic bacteria density, *r*_*s*_ = 0.89, *p* = 0.012)]. The measured concentrations of COD and BOD showed a strong correlation (*r*_*s*_ = 1, *p* > 0.001). All the sample locations exceed the total phosphorus threshold of 0.1 mg/L as well as the nitrogen threshold (1 mg/L). Temperature and nitrogen concentration were negatively correlated (*r*_*s*_ = −0.79, *p* = 0.048). Total phosphorus concentration was strongly negatively correlated with dissolved oxygen concentration (*r*_*s*_ = −0.96, *p* = 0.003). Literature classifies wastewater TSS as follows: TSS less than 100 mg/L as weak, TSS greater than 100 mg/L but less than 220 mg/L as medium and TSS greater than 220 mg/L as strong wastewater. Results of this study classified the sample locations located near to wastewater treatment plant effluent as weakly contaminated wastewater, which reflects the efficiency of wastewater treatment. Less contaminated sample locations close to open space were classified as medium wastewater. In total, 27 sample locations were classified as polluted sites and only three as less polluted. Locations number three (wastewater treatment plant effluent), 15 (slaughterhouse) and 25 (urban runoff) were categorized as less polluted sites in base of their physico-chemical results (Figure 1).

### Bacteriological analysis

**Figure 3:**
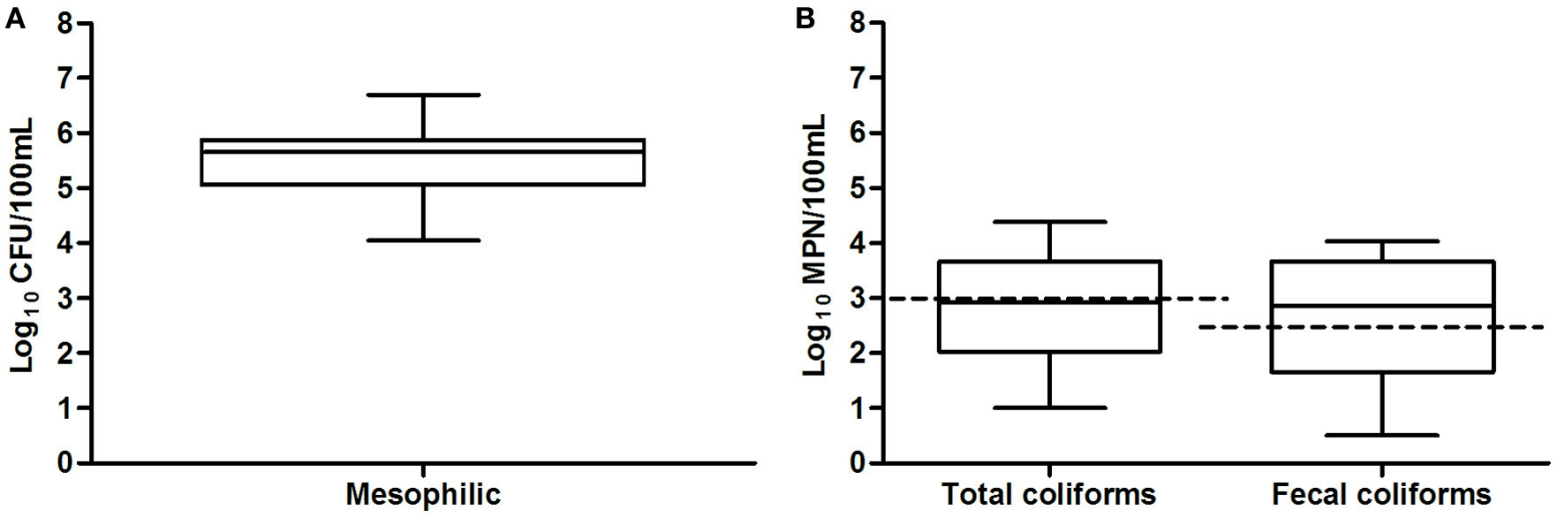
**Box and whiskers plot of results of microbiological analyses of San Pedro River in thirty sample location**. Bacterial counts values are shown in a log_10_ scale, **(A)** mesophilic microorganisms **(B)** total and fecal coliforms. Permissible maximum limits are shown in dotted lines. Total and fecal coliform density rating above 1000 MPN/100 mL and 240 MPN/100 mL are considered as poor water quality according to Mexican norms NOM-003-ECOL-1997 and NOM-001-ECOL-1996.

The mesophilic microorganism counts were between 10^4^ and 10^6^ CFU and these measurements were consistent with the high levels of organic and inorganic nutrients found in the San Pedro River, and the favorable physicochemical conditions for microbial growth found in the river (Figure 3). Although agricultural, farm and industrial sewage site locations tended to have greater counts of mesophilic bacteria than open space, no significative differences were found (*p* > 0.05). Half of the samples exceeded the limit of 1000 MPN/100 mL (WHO, 2006). Some samples were as low as 1 MPN and others as high as 2.4 × 10^4^ MNP/100 mL. Some samples presented low fecal coliforms counts as low as 0.5 MPN and others were as high as 1 × 10^4^ MNP/100 mL. Statistically significant associations were found between the levels of total and fecal coliforms and water temperature (*p* = 0.02), and between coliforms and conductivity (*p* = 0.03), suggesting fecal bacteria proliferation due to appropriate conditions in the water environment. Total and fecal coliform were strongly correlated (*r*_*s*_ = 0.86, *p* = 0.023). Industrial sewage and urban runoff sites tended to have greater total and fecal coliform densities than the agricultural, farm locations and wastewater treatment plant effluent.

### Antibiotic resistance phenotypes of *E. coli* isolates

**Figure 4:**
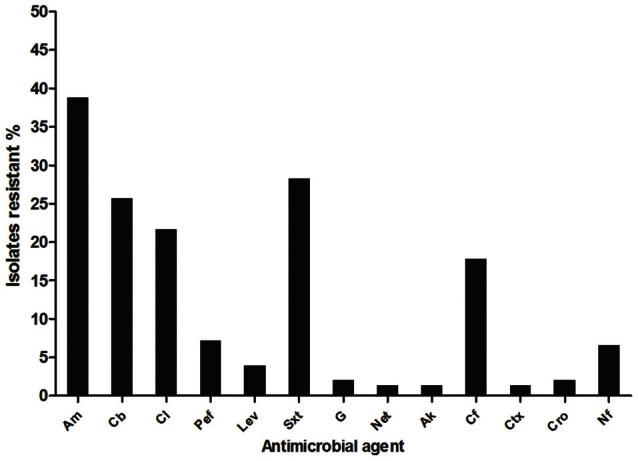
**Antimicrobial resistance (%) of *E. coli* isolated from San Pedro River**. A total of 150 isolates were characterized by antimicrobial susceptibility assay. ^a^Am, ampicillin; Cb, carbenicillin; Cl, chloramphenicol; Pef, pefloxacin; Lev, levofloxacin; Sxt, trimethoprim-sulfamethoxazole; G, gentamicin; Net, netilmicin; Ak, amikacin; Cf, Cephalotine; Ctx, cephatoxim; Cro, ceftriaxone; Nf, nitrofuratoin.

The antimicrobial susceptibility of 150 *E. coli* isolated from the San Pedro River to 13 antimicrobial agents was measured by the disc diffusion method (CLSI, 2010). Fifty-two percent (79/150) of the isolates were resistant to at least one antimicrobial agent; 37.3% (56/150) were resistant to at least two and 30.6% (46/150) were multi-drug-resistant. A total of 59 isolates (39.3%) were resistant to ampicillin (Figure 4). The second most prevalent antibiotic resistance was toward trimethoprim-sulfamethoxazole (28.6%, 43/150 isolates), followed by carbenicillin (26%, 39/150 isolates), chloramphenicol (22%, 33/150 isolates), and cephalothine (17.3%, 26/150 isolates). Few isolates (1.33%; 2/150) had a resistance toward cephotaxim, netilmicin and amikacin. Interestingly, it was noticed that 7.3% (11/150) of the isolates were resistant to pefloxacin and 4% (6/150 isolates) were resistant to levofloxacin (Figure 4). These antibiotics are second and third-generation quinolones widely used in Mexico against intestinal and UTIs (Guajardo-Lara et al., 2009). Furthermore, 12 strains were FQ-resistant and presented a multi-resistant phenotype. Most *E. coli* isolates with resistance to FQ were found in the sample locations close to farms, agricultural areas, urban runoff and industrial sewage.

**Figure 5:**
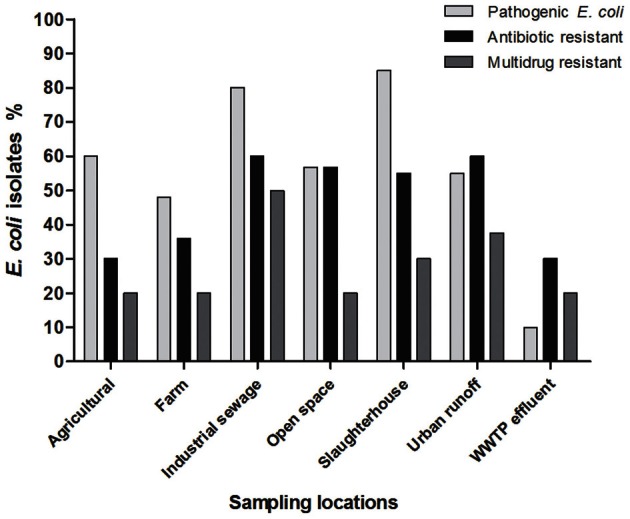
**Antimicrobial resistant, multidrug resistant, and potentially pathogenic *Escherichia coli* isolates found according to the sample location**. aWWTP-E, wastewater treatment plant effluent.

**Figure A1:**
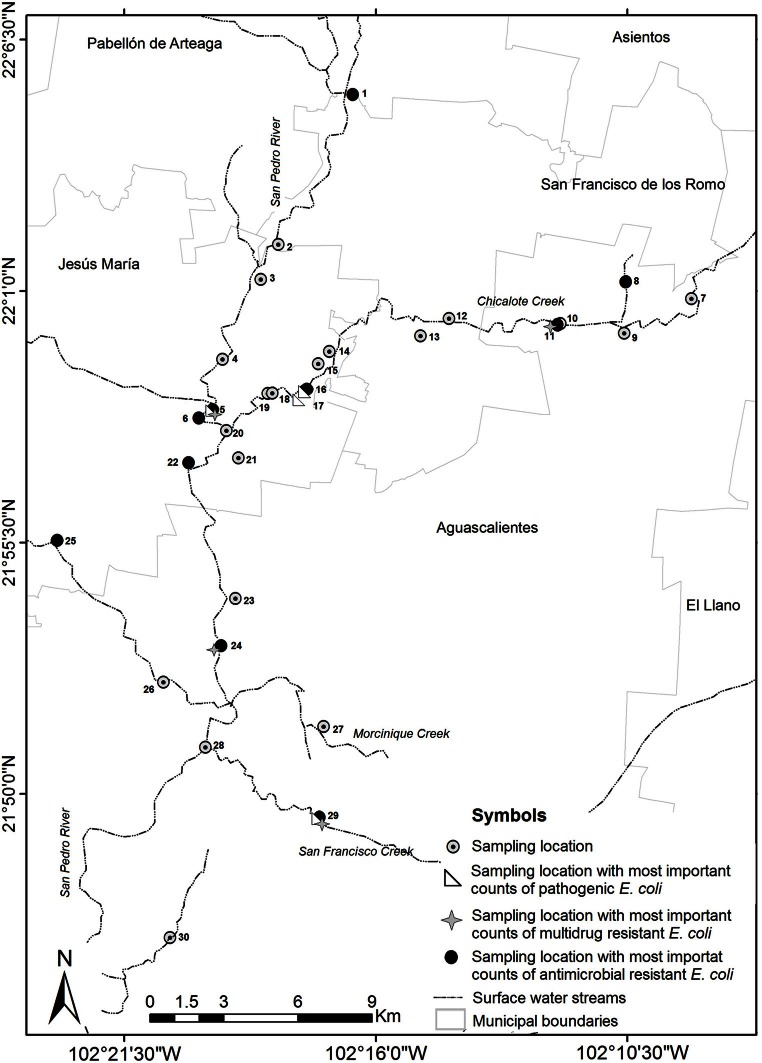
**Diagram showing the highest levels of pollution, highest counts of the pathotype, and antimicrobial resistant bacteria of the sample locations studied**.

Among isolates with a multi-resistant phenotype, 1.3% (2/150) was resistant to seven antimicrobial agents; 3.3% (5/150) were resistant to six antimicrobial agents; 5.3% (8/150) were resistant to five antimicrobial agents, 7.3% (11/150) were resistant to four antimicrobial agents and 13% (20/150) were resistant to three antimicrobial agents. The location sites close to discharges from urban runoff and industrial sewage had a more important proportion of isolates that were resistant to multiple antibiotics (15 isolates and 10 isolates respectively, Figure 5). Wastewater treatment plant effluent and agricultural locations had the lowest proportion of antibiotic resistant bacteria with only two multiresistant and one resistant bacteria in each location. Urban runoff locations as well as industrial sewage, open space and slaughterhouse sample locations had the most important counts of antibiotic resistant bacteria. Urban runoff sample locations were also the locations with highest density of total and fecal coliforms. Additionally, even when farm locations presented low proportion of antibiotic resistant bacteria, these locations presented multidrug resistance patters to more antibiotic classes (Figure A1). Non-resistant bacteria were found in samples isolates from farms (site locations No. 8 and 30), urban runoff (site location No. 14) and agricultural (site location No. 21) locations (Figure 5). Furthermore, density of multiresistant bacteria was negatively-correlated with nitrogen concentration (*r*_*s*_ = −0.78, *p* = 0.04).

A noticeable result shows that a co-resistance to beta-lactams and sulfonamides was frequently observed because most of sulfonamide-resistant isolates were also resistant to beta-lactams (Table 5). Resistance phenotype to quinolones was associated with beta-lactams and sulfonamides resistance (0.05 ≥ *p* ≥ 0.01), and beta-lactams and phenicols resistance (0.01 ≥ *p* ≥ 0.001).

Wastewater treatment plant effluent sample, which was considered as less polluted based on tested parameters (Figures 2, 3) presented proportion of antibiotic resistant bacteria similar to that of the agricultural sector (30%). Nevertheless, samples from industrial sewage had the highest proportion of multidrug resistant bacteria (50%) suggesting that industrial sewage might have an impact on the presence of multidrug resistant bacteria.

### Pathotype and virulence genes determination of *E. coli* isolates

Sixty percent (91/150) of the strains were PCR positive for at least one virulence gene (Aranda et al., 2007). Eighty-six isolates were identified as InPEC, including 44.6% (67/150) EAEC, 6.6% (10/150) EPEC, and 6% (9/150) ETEC (Table 4). Only 5 (3.3%) isolates were identified as incomplete ExPEC because these isolates were positive for the virulence genes *fyuA*, *kpsMII*, *sfa*, and *afa/dra* (Johnson and Stell, 2000). EIEC and Shiga-toxigenic *E. coli* were not detected. In addition, slaughterhouse (85%, 17/20 isolates), industrial sewage (80%, 16/22 isolates) and agricultural (60%, 6/10 isolates) sample locations had the most important proportion of pathogenic bacteria.

**Table 4:** **Antimicrobial resistance found in potentially pathogenic and commensal *Escherichia coli***.

**Antimicrobial class**	**Antimicrobial agent**	**No. (%)^a^ of pathogenic *E. coli*^b^**				**Commensal (*n* = 59)**
		**EPEC (*n* = 10)**	**ETEC (*n* = 9)**	**EAEC (*n* = 67)**	**Incomplete ExPEC (*n* = 5)**	
Aminoglycosides	Gentamicin	0 (0)	0 (0)	3 (4)	0 (0)	0 (0)
	Netilmicin	0 (0)	0 (0)	1 (1)	0 (0)	1 (2)
	Amikacin	0 (0)	0 (0)	0 (0)	0 (0)	2 (3)
Phenicols	Chloramphenicol	3 (30)	3 (33)	16 (24)	1 (20)	10 (17)
Quinolones	Pefloxacin	1 (10)	1 (11)	3 (4)	0 (0)	7 (12)
	Levofloxacin	1 (10)	1 (11)	3 (4)	1 (20)	0 (0)
Sulfonamides	Trimethoprim-sulfamethoxazole	3 (30)	3 (33)	22 (33)	0 (0)	14 (24)
Beta-lactams	Ampicillin	6 (60)	4 (44)	29 (43)	1 (20)	17 (29)
	Carbenicillin	5 (50)	3 (33)	19 (28)	1 (20)	12 (20)
Nitrofurans	Nitrofuratoin	1 (10)	0 (0)	5 (7)	1 (20)	3 (5)
Cephalosporins	Cephalotine	2 (20)	1 (11)	12 (18)	0 (0)	7 (12)
	Cephatoxim	1 (10)	0 (0)	1 (1)	0 (0)	0 (0)
	Ceftriaxone	1 (10)	0 (0)	2 (3)	0 (0)	0 (0)

a Percentages were calculated as follows: number of isolates with resistance phenotype/total number of E. coli isolates per pathotype per 100.

b EPEC, enteropathogenic E. coli. ETEC, enterotoxigenic E. coli. EAEC, enteroaggregative E. coli. Incomplete ExPEC, incomplete extra-intestinal pathogen E. coli.

**Table 5:** **Association between antimicrobial resistance phenotypes of *E. coli* isolates from stream water**.

**Antimicrobial agent**		**Phenicols**	**Quinolones**		**Sulfonamides**	**Beta-lactams**		**Cephalosporin**
		**Chloramphenicol**	**Pefloxacin**	**Levofloxacin**	**Trimethoprim-sulfamethoxazole**	**Ampicillin**	**Carbenicillin**	**Cephalotine**
Quinolones	Pefloxacin	++						
	Levofloxacin	−	+					
Sulfonamides	Trimethoprim-sulfamethoxazole	++	++	++				
Beta-lactams	Ampicillin	++	+++	++	−			
	Carbenicillin	++	++	−	+	+		
Nitrofurans	Nitrofuratoin	+	+	−	−	−	+++	
	Cephalotine	−	+++	++	+++	+++	+++	
Cephalosporin	Cephatoxim	−	−		−	+	−	−
	Ceftriaxone	++	−	−	−	++	−	+

Only the antimicrobial multi-resistant phenotypes that exhibited an association with another phenotype at the p < 0.05 level are shown. The levels of significance of the association as assessed by the chi-square exact test were as follows: –, p > 0.05; +, 0.05 ≥ p ≥ 0.01; ++, 0.01 ≥ p ≥ 0.001; +++, 0.001 ≥ p.

Furthermore, strains belonging to the pathotypes EPEC (*n* = 7), ETEC (*n* = 4), EAEC (*n* = 37) and incomplete ExPEC (*n* = 4) were at least resistant to one antimicrobial agent. Among strains with a multi-antimicrobial resistance phenotype, 22 were EAEC, 4 were ETEC isolates, 6 were EPEC, and 1 was an incomplete ExPEC. Most pathogenic *E. coli* that had multi-resistant phenotype were resistant to beta-lactams and trimethoprim-sulfamethoxazole. Amongst the commensal *E. coli*, multi-resistance was also detected in 22% (13/59) of the isolates.

Significative differences between number of pathogenic isolates (*p* = 0.02) and isolates with resistance to one antimicrobial agent (*p* = 0.04) were found when comparing polluted samples with that of less polluted sample sites. Non-statistical differences were found compared multidrug resistance (*p* > 0.05). This suggests that in polluted water there are more pathogenic bacteria (Figure 5).

### Characterization of quinolone resistance in *E. coli* isolates

The genotypes associated with the quinolone resistance (including intermediate resistance) phenotype were characterized for seven pathogenic *E. coli* and 11 commensal *E. coli*. Three of 18 isolates were resistant to second generation quinolones (levofloxacin and pefloxacin), nine isolates were resistant to one of the two quinolones and six showed intermediate resistance to pefloxacin (6/18 isolates). Resistance to quinolones was usually observed in strains with a multi-drug resistance phenotype (16/18 isolates).

The sequencing results for the QRDR of *gyrA* and *parC* are summarized in Table 6. The Ser-83 → Leu and Asp-87 → Asn substitution in *gyrA* and the Ser-80 → Ile substitution in *parC* were found in isolates resistant to both levofloxacin and pefloxacin (*n* = 2), to levofloxacin alone (*n* = 2), and to pefloxacin alone (*n* = 3). *E. coli* isolates with only the Ser-83 → Leu and Asp-87 → Asn substitution in *gyrA* showed resistance and intermediate resistance to pefloxacin. A single mutation in *gyrA* at Ser-83 → Leu (*n* = 2) was found in one strain resistant to both (levofloxacin and pefloxacin), and one with intermediate resistance to pefloxacin. An isolates with a single mutation (Ser-80 → Ile) in *parC* exhibited an intermediate resistance toward pefloxacine. Three strains had one or more *qnr* genes. Five *E. coli* isolates possessed *qnrA*, seven isolates had *qnrS* and two isolates had *qnrB*. Overall, eight isolates had chromosomal mutations in *gyrA*, *parC* or both as well as horizontally acquired *qnr* genes. The *qnr* genes were found in two strains with a triple mutation profile (Ser-83 → Leu, Asp-87 → Asn in *gyrA* and Ser-80 → Ile in *parC*) that were resistant to both levofloxacin and pefloxacin. Quinolone resistance isolates (*n* = 3) with one mutation Ser-83 → Leu in *gyrA*, or, Ser-80 → Ile in *parC* possessed also *qnr* genes. The three strains with no chromosomal mutation possessed one or two *qnr* genes. One of the isolates did not have either a chromosomal mutations or *qnr* genes. In addition, the ETEC pathotype isolate 18A (Table 6) exhibited a multi-resistance phenotype and had three substitutions (Ser-83 → Leu, Asp-87 → Asn in *gyrA* and Ser-80 → Ile in *parC*), *qnrS* and the newly described *aac (6′)-Ib-cr* gene. Additionally, five isolates that had the triple mutation profile (Ser-83 → Leu, Asp-87 → Asn in *gyrA* and Ser-80 → Ile in *parC*) in the QRDR carried also one *qnr* gene (*qnrA* or *qnrS*) and at least one beta-lactamase gene.

**Table 6:** **Summary of the pathotype, antimicrobial resistance patterns, QRDR mutation and presence of *qnr* resistance genes for the 18 *E. coli* isolates selected for their resistance to fluoroquinolones of second (pefloxacin) and third (levofloxacin) generation**.

**Strains ID**	**MDR phenotype^a^**	**Diffusion disc^b^**		**QRDR mutation**		**PMQR genes**	**Other resistance genes**	***E. coli* type^c^**
		**LEV**	**PEF**	**→GyrA**	**→ParC**			
16C	AmCbClSxtLevPef	R	R	S83→L, D87→N	S80→I	*qnrA*	*aph3(strA)*, *bla*_TEM_, class 1 integron	Incomplete ExPEC
18A	AmCfCtxCroLevPefStx	R	R	S83→L, D87→N	S80→I	*aac-(6′)-lb-cr*, * qnrS*	*aph3(strA)*, *aph6(strB)*, *bla*_TEM_, *tet(B)*, *tet(M)*	InPEC
18C	AmCfCtxCroLevPefStx	R	R	S83→L	None	*aac-(6′)-lb-cr*, * qnrS*	*aph3(strA)*, *aph6(strB)*, *bla*_TEM_, * dhfrVII*, *tet(B)*	InPEC
17A	AmGLevSxt	R	S	S83→L, D87→N	S80→I	None	*bla*_TEM_, *aac(3)-IIa(aacC2)*, *aph3(strA)*, *mphA*, *sulII*, class 1 and 2 integron	InPEC
17D	AmCbGLev	R	S	S83→L, D87→N	S80→I	None	*aph3(strA)*, *bla*_TEM_, *aac(3)-IIa(aacC2)*, *mphA*, *sulI*, *sulII*, *tet(B)*, class 1 integron	InPEC
17E	AmCbLev	R	S	None	None	None	*aph3(strA)*, *bla*_TEM_, *dhfrXII*, *sulII*, *tet(A)*	Incomplete ExPEC
24A	AmCbClCfPefSxt	S	R	S83→L, D87→N	S80→I	*qnrA*	*aph3(strA)*, *bla*_TEM_, *dhfrXII*, *mphA*, *sulII*, *tet(B)*, class 1 integron	Commensal
16D	AmCbClPefSxt	S	R	S83→L, D87→N	S80→I	*qnrA*	*aph3(strA)*, *bla*_PSE_, *bla*_TEM_, *catI*, *dhfrXII*, *mphA*, *tet(A)*, *tet(B)*, *sulI*, *sulII*, class 1 and 2 integron	Commensal
24C	AmCbClPefSxt	S	R	S83→L, D87→N	S80→I	None	*cmIAI*, *dhfrXII*, class 1 integron	Commensal
24E	AmCbClPefSxt	S	R	S83→L, D87→N	S80→I	None	*dhfrXII*, *cmlAI*.	Commensal
1A	CfNfPef	S	R	S83→L, D87→N	None	*qnrS*	*aph3(strA)*	Commensal
22E	Pef	S	R	None	None	*qnrS*	ND	Commensal
3A	AmClStxPef	S	I	None	None	*qnrA*	*aph3(strA)*, *bla*_PSE_, * dhfrVII*, *sulII*, *tet(A)*, class 2 integron	InPEC
2E	AmClStxPef	S	I	S83→L, D87→N	None	None	Class 1 and 2 integron.	Commensal
29A	AmCbNfSxtPef	S	I	None	S80→I	*qnrA*, *qnrB*	*bla*_PSE_, *dhfrXII*, class 1 integron	InPEC
20A	AmPef	S	I	None	None	*qnrB*, *qnrS*	*aph3(strA)*, *bla*_TEM_	Commensal
29E	AmCfCbSxtPef	S	I	None	None	*qnrS*	*aadA (1)*, *bla*_TEM_, *dhfrI*, *sulI*, class 1and 2 integron, transposon *Tn21*	InPEC
25A	AmCbPef	S	I	S83→L	None	*qnrB*, *qnrS*	Transposon *Tn21*	Commensal

a Multi-drug resistance (MDR) phenotype, Am, ampicillin; Cb, carbenicillin; Cl, chloramphenicol; Pef, pefloxacin; Lev, levofloxacin; Sxt, trimethoprim-sulfamethoxazole; G, gentamicin; Net, netilmicin; Ak, amikacin; Cf, Cephalotine; Ctx, cephatoxim; Cro, ceftriaxone; Nf, nitrofuratoin. ND, not determined.

b R, resistant; I, intermediate resistance; S, susceptible.

c InPEC, intestinal E. coli; ExPEC, extraintestinal E. coli.

### Virulence and antimicrobial resistance genes among quinolone resistant *E. coli* isolates

Microarray analysis was done on 17 quinolone resistant *E. coli* isolates. Based on the microarray analysis, nine isolates were classified as commensal and six were classified as InPEC, ExPEC, or potentially pathogenic *E. coli*. The isolates belonged to the phylotype group A (14 isolates), group D (1 isolate) and B1 (2 isolate). The most frequent antimicrobial resistance genes were *aph3strA* (10/17), *bla*_TEM_ (10/17) and *tet(B)* (5/17), which code for resistance to streptomycin, ampicillin, and tetracycline respectively (Table 6). Class 1 integron markers were also found in ten isolates. A *bla*_TEM_ and *aph3(strA)* combination was observed in nine isolates, and a *bla*_TEM_, *aph3(strA)* and *tet(B)* combination was observed in six isolates. In our study, 10 quinolone resistance isolates also carried *bla*_TEM_ gene. Among these 10 isolates, nine isolates were positive for the beta-lactamase gene and a plasmid acquired resistance gene.

Isolates with *qnr* genes and the triple mutation patter in the QRDR were found in sample locations close to farm and agricultural sector (Figure 1, Table 6). Nevertheless, isolates with intermediate-resistance to FQ phenotypes were found in sample locations close to urban runoff, industrial sewage, slaughterhouses, open space and wastewater treatment plant. Interestingly, samples with resistance to one or other FQ phenotype were sampled in locations close to urban runoff, industrial sewage, open space, farm, and agricultural sectors (Table 6, Figures 1, 5).

## Discussion

Aquatic environments such as rivers and streams are considered ideal reservoirs for antibiotic resistance dissemination, since antimicrobials and antimicrobial resistant bacteria are often directly released in the environment (Roe et al., 2003; Zhang et al., 2009b; Lupo et al., 2012). In developing countries, the contribution of sewage and wastewater as point sources, as well as animal farming without adequate outlet control, represent extra loads of contamination for aquatic systems. Most of the water from these activities is subsequently used for irrigation, without any treatment. This can represent potential risks of contamination (Mazari-Hiriart et al., 2008; Zhang et al., 2009c; Jang et al., 2013). In the State of Aguascalientes in Mexico, this is of great importance because river water is directly used for irrigation and in rural communities as a source of drinking water for livestock. Our results showed that the concentration of coliforms in San Pedro River and its major creeks was exceeding by more than one order of magnitude the WHO tolerance limits (Figure 3). The physicochemical values and microbiological counts found in the San Pedro River are consistent with an important pollution originating from municipal and livestock wastewater (Figures 1, 2). Furthermore, these conditions provide a favorable environment for microbial growth (Figures 2, 3). Given that the water from the San Pedro River is used for irrigation, the water may constitute a source of bacterial contamination that could infect humans or animal through direct contact, aerosol, or consumption of vegetables.

In Mexico, antibiotics are medical drugs with high rate of consumption and their consumption is associated with a high rate of misuse (Dreser et al., 2008). Misuse is caused by unwarranted prescription, inappropriate choice of treatment, self-prescription, lack of adherence by consumers, as well as lax regulation on the use of antibiotics (Zaidi et al., 2003; Amábile-Cuevas et al., 2010). The misuse and overuse of antibiotics is particularly important because it may contribute to selection and increased occurrence of antimicrobial resistant bacteria.

Anthropogenic-driven selective pressures may be contributing to the persistence and dissemination of genes and antimicrobial resistant bacteria usually relevant in clinical environments (Tacão et al., 2012). Our results indicated that samples from industrial and urban runoff sewage had an important presence of antibiotic resistance bacteria (Figure 5). Moreover, the highest number of multi-resistant isolates was found in samples from wastewater discharges from human sewage, industrial sector, and farms. This suggests the importance of wastewater discharges in the dissemination of antimicrobial resistance strains. In the multi-resistant isolates, resistance to FQs such as pefloxacin and levofloxacin was significatively associated with resistance to trimethoprim-sulfamethoxazol and ampicillin (*p* < 0.05). This association is likely due to the presence of *qnr* genes and beta-lactams resistance genes in the multi-resistant isolates (Wang et al., 2001).

At least two thirds of all *E. coli* isolates were resistant to beta-lactams such as ampicillin and carbenicillin, and at least one third of the isolates were resistant to trimethoprim-sulfamethoxazole and chloramphenicol (Figure 4). The presence of antibiotic resistant *E. coli* was also observed in other studies from human and animal fecal sources, wastewater treatment plant and surface water (Sayah et al., 2005; Ibekwe et al., 2011; Mokracka et al., 2011; Sun et al., 2012). Similar resistance levels were found in *E. coli* isolated from children and adults in Latin America and the 53.2 and 57.7% isolates were resistant to ampicillin and trimethoprim-sulfamethoxazol, respectively (Estrada-Garcia et al., 2005, 2009).

Most *E. coli* identified as of pathogenic or potentially pathogenic were classified as intestinal pathogens (61%, 91/150). EAEC was the most prevalent intestinal pathogenic *E. coli* (44% of the isolates), and this is consistent with previous studies conducted in Mexico (Estrada-Garcia et al., 2005), since in clinical settings, EAEC and ETEC are the most prevalent pathotypes in Mexico (Estrada-Garcia et al., 2005, 2009). Furthermore, several intestinal pathogens with multiple-resistance to antimicrobials were isolated from the River. Thus, the occurrence of pathogenic *E. coli* with multiple antimicrobial resistances in the San Pedro River represents a great concern due to possible transfer of resistant genes and may increase the probability of infections with a higher cost of treatment.

In our study, most of the isolates resistant to levofloxacin and pefloxacin had a multi-resistant phenotype and some were potentially pathogenic *E. coli*. Similar results were found in a Mexican study on the prevalence of FQ resistance among *E. coli* isolates from urinary tract infection (Zaidi et al., 2003; Llanes et al., 2012) as well as from an environmental study (Amábile-Cuevas et al., 2010). In addition, we showed that the triple mutation profile (Ser-83 → Leu, Asp-87 → Asn in *gyrA*, and Ser-80 → Ile in *parC*) was the most prevalent. The point mutations Ser-83 → Leu and Asp-87 → Asn found in *gyrA* and Ser-80 → Ile in *parC*, have been observed in other studies (Namboodiri et al., 2011; Sun et al., 2012). Previous studies have shown that *E. coli* with a single mutation (Ser-83 → Leu) in the *gyrA* subunit are resistant to nalidixic acid, a first generation quinolone (Vila et al., 1994; Sun et al., 2012). In addition, most fluoroquinolone resistance isolates carried horizontally acquired quinolone resistance genes and these were found primarily in combination with QRDR mutations. Qnr proteins may supplement resistance to quinolones due to altered quinolone target enzyme, efflux pump activation, or deficiencies in outer-membrane porins (Martinez-Martinez et al., 2003; Poirel et al., 2012). The presence of Qnr determinants facilitates the selection of low-level of resistance to quinolones encoded on the chromosome-encoded and the selection of higher-level resistance mutation (Jacoby, 2005). Several strains that harbored *qnrS* also carried *bla*_TEM_ gene. It was reported that, *qnrS* genes are associated with transposons containing TEM-1 type -lactamases (Hernandez et al., 2011; Dalhoff, 2012). Tetracycline and streptomycin resistance genes were also detected along with FQ resistance genes (Roberts, 2005; Zhang et al., 2009a).

Our results revealed the presence of pathogenic *E. coli* in the river with present mobile elements as integrons, and multidrug resistance characteristics, including FQ resistance, an antibiotic highly used in humans and animals worldwide, mostly found in locations of the river that have been impacted by industrial sewage and urban runoff. This situation highlights the risk of multidrug resistance pathogens dissemination (Allou et al., 2009; Dalhoff, 2012; Sun et al., 2012). Furthermore, our study was conducted in a densely populated area, a setting that is often encountered in developing countries and that must be taken into account (Mazari-Hiriart et al., 2008). This poses a potential risk for human infections because water is used for consumption or for recreation (Maynard et al., 2005; Hamelin et al., 2006, 2007).

The permanent influx of pollutants such as antimicrobial agents, detergents, disinfectants, heavy metals, livestock waste, and watershed may contribute to the emergence of antibiotic resistant bacteria in water as well as the spread of antimicrobial resistance genes and virulence bacteria in San Pedro River. The results show that it is urgent to evaluate the management of wastewater and the water quality in San Pedro River and if necessary, implement a local wastewater treatment to prevent the emergence of infectious outbreaks.

### Conflict of interest statement

The authors declare that the research was conducted in the absence of any commercial or financial relationships that could be construed as a potential conflict of interest.
